# Unbalance of intestinal microbiota in atopic children

**DOI:** 10.1186/1471-2180-12-95

**Published:** 2012-06-06

**Authors:** Marco Candela, Simone Rampelli, Silvia Turroni, Marco Severgnini, Clarissa Consolandi, Gianluca De Bellis, Riccardo Masetti, Giampaolo Ricci, Andrea Pession, Patrizia Brigidi

**Affiliations:** 1 Department of Pharmaceutical Sciences, University of Bologna, Via Belmeloro 6, Bologna, 40126, Italy; 2 Institute of Biomedical Technologies, Italian National Research Council, Milan, Italy; 3 Paediatric Oncology and Haematology Unit Lalla Seràgnoli, Sant’Orsola-Malpighi Hospital, University of Bologna, Bologna, Italy; 4 Department of Gynecological, Obstetrical and Pediatric Sciences, Sant’Orsola-Malpighi Hospital, University of Bologna, Bologna, Italy

## Abstract

**Background:**

Playing a strategic role in the host immune function, the intestinal microbiota has been recently hypothesized to be involved in the etiology of atopy. In order to investigate the gastrointestinal microbial ecology of atopic disease, here we performed a pilot comparative molecular analysis of the faecal microbiota in atopic children and healthy controls.

**Results:**

Nineteen atopic children and 12 healthy controls aged 4–14 years were enrolled. Stools were collected and the faecal microbiota was characterized by means of the already developed phylogenetic microarray platform, HTF-Microbi.Array, and quantitative PCR. The intestinal microbiota of atopic children showed a significant depletion in members of the *Clostridium* cluster IV, *Faecalibacterium prausnitzii*, *Akkermansia muciniphila* and a corresponding increase of the relative abundance of *Enterobacteriaceae.*

**Conclusion:**

Depleted in key immunomodulatory symbionts, the atopy-associated microbiota can represent an inflammogenic microbial consortium which can contribute to the severity of the disease. Our data open the way to the therapeutic manipulation of the intestinal microbiota in the treatment of atopy by means of pharmaceutical probiotics.

## Background

Atopic diseases are chronic inflammatory disorders caused by an aberrant T-helper (Th2)-type immune response against common and innocuous environmental antigens [1]. The elaboration of cytokines, such as interleukin (IL)-4, IL-13 and IL-5, can contribute to disease induction [2]. During the past decades the prevalence of atopic diseases among children in the western world has dramatically increased [2]. Too fast for any possible shift in genetic constitution, environmental changes associated with the western lifestyle are believed to be involved [3,4]. The human intestinal microbial community has been indicated as a key factor to interpret the impact of the western lifestyle on the etiology of atopic diseases [3-7]. The intestinal microbiota is extremely plastic in response to diet and environmental factors and, at the same time, governs many aspects of the immune function throughout the body [8]. Thus, the hypothesis that specific western lifestyle-driven dysbioses of the human intestinal microbiota are involved in the bloom of atopy in western children has been advanced.

The human intestinal microbiota consists of a total amount of 10^14^ microbial cells differentiated in more than 500 different species [9]. Showing a tremendous metabolic potential, this versatile microbial “organ” exerts a role of primary importance for our metabolism. Recently, the strategic role of the intestinal microbiota in the development, education and functionality of the human innate and adaptive immune system has been recognized [7,10]. According to Gaboriau-Routhiau *et al.*[11], specific members of the intestinal microbial community exert an active role in the modulation of a striking range of T cell functions, such as Th17, Th1, Th2 and regulatory cell phenotype (T regs). Having a profound impact on the overall human immune status, perturbations of the intestinal microbiota have been implicated in the development and progression of inflammatory diseases, such as inflammatory bowel diseases (IBD), autoimmune disorders, allergy and type II diabetes [12,13].

On the basis of the perceived importance of the intestinal microbiota in the education of the human immune system to tolerance [5], culture-independent perspective studies have been carried out to determine whether specific microbiota dysbioses in the early life could affect the subsequent manifestation and sensitization of atopic diseases. In the Lifestyle and Genetic Constitution (KOALA) Birth Cohort Study – an extensive epidemiological study with involved 957 infants from Netherlands aged 1 month – the presence of *Escherichia coli* and *Clostridium difficile* in stools has been associated with a higher risk to develop eczema [14]. Even if the health-promoting microbiota components *Bifidobacterium* and *Lactobacillus* have been suggested as possible protective factors against the risk to develop atopy [15,16], no differences in the prevalence of these probiotic genera between infants with and without allergic disorders have been detected [3,14,17,18]. More recently, two perspective surveys of the intestinal microbiota in Danish and Swedish infants have been carried out with a longitudinal approach, sampling the faecal microbiota at different time points during the first year of life [19,20]. Based on denaturing gradient gel electrophoresis (DGGE) and 16S rDNA 454-pyrosequencing, respectively, these robust and extensive studies proved that the low bacterial diversity in the early life, rather than the prevalence of a specific bacterial taxon, is associated with an increased risk of subsequent atopic disease, reinforcing the “old friend hypothesis” [21]. According to this theory, the western lifestyle caused the disappearance of key bacterial groups from the intestinal microbiota, which are essential to prime the physiology of our immune system. The lack of these “old friends” during the perinatal period led to an immune system incline to inappropriate activation, which is a characteristic of the emerging chronic inflammatory diseases in the western world.

Even if the role of the intestinal microbiota in the predisposition to develop atopy in infancy has been accepted, to our knowledge, only few case–control culture-independent studies of the gastrointestinal microbial ecology in atopic diseases have been carried out. However, by modulating the immune status throughout the body [8], an inflammogenic gut microbial community in atopic subjects could significantly contribute to the severity of the disease. In this perspective we performed a pilot case–control study of the atopy-associated dysbiosis of the intestinal microbiota in atopic children. Since from birth to weaning the infant intestinal microbiota is an extremely dynamic entity, which continuously fluctuates in response to factors of environmental and endogenous origin [22], we enrolled children aged > 2 years, characterized by a relatively stable adult-like intestinal microbial community [23]. In particular, the faecal microbiota of 19 atopic children and 12 healthy controls aged 4–14 years was characterized by means of the previously developed phylogenetic microarray platform High Taxonomic Fingerprint (HTF)-Microbi.Array [24] and quantitative PCR (qPCR). Integrated of an additional probe pair for *Akkermansia muciniphila*, the HTF-Microbi.Array platform detects up to 31 intestinal bacterial groups and covers up to 95% of the human intestinal microbiota [25]. For our study faeces were selected since they represent the only realistic and reliable sample for a non-invasive study of the human intestinal microbiota.

## Methods

### Subjects enrolled and study groups

We enrolled 19 children (referred as atopics throughout the paper) with clinical diagnosis of allergy (rhinitis, asthma, grass pollen sensitization, allergic atopic dermatitis, oral allergy syndrome, cow’s milk allergy) and encountering all the following criteria: (i) delivered naturally at term, (ii) breast fed for at least 3 months, (iii) aged between 4 and 14 years, (iv) no acute diseases for at least 2 weeks, (v) no antibiotic treatment in the last 3 months. In particular, 17 children presented allergic rhinitis, in 4 cases associated with asthma. Atopic dermatitis was observed in 8 cases of which 6 associated with rhinitis and inhalant sensitization and 1 with food allergy (Table 1). During the visit the children underwent a clinical evaluation and skin prick test for main food or inhalant allergens. Total and specific IgE determination was performed when clinically necessary. Fresh stool samples were collected within 3 days. As controls, 12 non-allergic children who encountered the same criteria above described but without family history of atopy were enrolled. All the children were routinely followed by the Paediatric Oncology and Haematology Unit Lalla Seràgnoli, Sant’Orsola-Malpighi Hospital, University of Bologna. Parents provided a written informed consent. Approval by the Ethics Committee of the Sant’Orsola-Malpighi Hospital was not needed for this study.

**Table 1:** Allergic profile of the 19 atopic children enrolled in the study

	**Clinical diagnosis of allergy**					
**Sample ID**	**RC**^**a**^	**A**^**b**^	**GPS**^**c**^	**AD**^**d**^	**OAS**^**e**^	**CMA**^**f**^
**A1**	√		√	√	√	
**A2**	√		√	√		
**A3**	√		√			
**A4**	√	√	√			
**A5**	√	√	√			
**A6**	√		√		√	√
**A7**	√		√	√		
**A8**	√		√	√		
**A9**	√	√	√		√	√
**A10**	√	√	√			
**A12**	√		√	√		
**A13**	√		√	√		
**A14**	√		√			
**A15**				√		
**A17**	√		√			
**A19**	√		√			
**A20**		√	√			
**A21**	√			√		
**A22**	√		√			

^a^Rhinitis.

^b^Asthma.

^c^Grass Pollen Sensitization.

^d^Allergic Atopic Dermatitis.

^e^Oral Allergy Syndrome.

^f^Cow’s Milk Allergy.

### Allergometric tests

Skin prick tests were performed following established guidelines [26]. The following allergens were tested: cow’s milk, egg, soy bean, wheat, peanut, codfish, grass pollen, *Dermatophagoides pteronyssinusDermatophagoides farinae*, and cat dander. Other allergens were tested on the basis of the child’s history. Data of the skin prick tests were used to determine the presence of atopic sensitization in the definition of allergic or non-allergic atopic dermatitis. The determination of total serum IgE was performed by ELISA test; the values were assumed as normal or increased in comparison with the ones from children of the same age group [27]. The determination of specific IgE was performed by UNICAP 1000 (Phadia) in all patients for the following allergens: cow’s milk, egg, soy bean, wheat, peanut, codfish, Bermuda grass, timothy grass, *D. pteronyssinusD. farinae*, and cat dander. Other allergens were tested on the basis of the child’s history.

### DNA extraction and polymerase chain reaction (PCR)

Total DNA from faecal material was extracted using QIAamp DNA Stool Mini Kit (Qiagen) according to the modified protocol reported by Candela *et al.*[24]. Final DNA concentration was determined using NanoDrop ND-1000 (NanoDrop Technologies). PCR amplifications were performed with Biometra Thermal Cycler T Gradient (Biometra). The 16 S rRNA gene was amplified using universal forward primer 27 F and reverse primer r1492, following the protocol described by Candela *et al.*[24]. PCR products were purified by using the Wizard SV gel and PCR clean-up System kit (Promega), eluted in 20 μl of sterile water and quantified with the DNA 7500 LabChip Assay kit and BioAnalyzer 2100 (Agilent Technologies). All the oligonucleotide primers used for PCR reactions and probe pairs employed for the array construction were synthesized by Thermo Electron.

### HTF-microbi.Array analysis

The HTF-Microbi.Array utilized in this study is based on the Ligase Detection Reaction-Universal Array (LDR-UA) approach [28] and enables specific detection and quantification of the 16 S rRNA from 31 phylogenetically related groups of the human intestinal microbiota (Additional file 1). The original HTF-Microbi.array [24] was updated to include a probe for the detection of *A. muciniphila*. The new probe was designed and validated as reported by Candela *et al.*[24] (Additional file 2). Sequences of the entire probe set of the HTF-Microbi.Array are reported in Additional file 3. Slide chemical treatment, array production, LDR protocol and hybridization conditions were carried out as previously reported [28,29] with probe annealing set at 60°C. The LDRs were carried out in a final volume of 20 μl with 50 fmol of PCR product. Two hundred and fifty fmol of synthetic template (5’-AGCCGCGAACACCACGATCGACCGGCGCGCGCAGCTGCAGCTTGCTCATG-3) were used for normalization purposes. All HTF-Microbi.Array experiments were performed in independent duplicates.

### Data analysis

All arrays were scanned and processed according to the protocol and parameters already described by Candela *et al.*[24]. Fluorescence intensities (IF) were normalized on the basis of the synthetic ligation control signal: (a) outlier values (2.5-fold above or below the average) were discarded; (b) a correction factor was calculated in order to set the average IF of the ligation control to 50000 (n = 6); (c) the correction factor was applied to both the probes and background IF values. Reproducibility of the experiments was assessed by calculating Pearson’s correlation of the fluorescence signals between the two replicates. LDR experiments showing a Pearson’s correlation coefficient <0.95 were repeated. Mean data from two replicated experiments were obtained and utilized for principal component analysis (PCA), box plot analysis and calculation of the probe relative IF contribution. Non-parametric Kruskal-Wallis test was used to determine whether the contribution of each bacterial group was significantly different between atopics and controls. Two-sided *t*-test was applied to evaluate whether the relative percentage contribution of each bacterial group was significantly different between the two groups. Correlation between variables was computed by Spearman rank correlation coefficient. Statistical analyses were performed by using Canoco package for Windows [30] and the R statistical software (http://www.r-project.org).

### Quantitative PCR

qPCR was carried out in a LightCycler instrument (Roche). Quantification of the 16 S rRNA gene of *A. muciniphila*, *Faecalibacterium prausnitzii*, *Enterobacteriaceae, Clostridium* cluster IV, *Bifidobacterium* and *Lactobacillus* group was performed with the genus-, group- or species-specific primers reported in Table 2. SYBR Green I fluorophore was used to correlate the amount of PCR product with the fluorescent signal. For quantification, standard curves were generated with known amounts of pCR2.1 (Invitrogen)-cloned 16 S rRNA gene from *A. muciniphila* (DSM22959), *F. prausnitzii* (DSM17677), *E. coli* (ATCC11105), *Clostridium leptum* (DSM753), *Bifidobacterium animalis* subsp. *lactis* (BI-07) and *Lactobacillus acidophilus* (LA-14). Amplification was carried out in a 20 μl final volume containing 100 ng of faecal DNA, 0.5 μM of each primer and 4 μl of LightCycler-FastStart DNA Master SYBR Green I (Roche). Amplifications were done under the following conditions: (i) starting preincubation at 95°C for 10 min; (ii) amplification including 35 cycles of 4 steps each at the temperature transition rate of 20°C/s: denaturation at 95°C for 15 s, annealing at the appropriate temperature (Table 2) for 20 s, extension at 72°C for 30 s, and fluorescence acquisition at the appropriate temperature (Table 2) for 5 s; (iii) melting curve analysis.

**Table 2:** **Primer sets used for the 16S rRNA gene quantification of*A. muciniphila*,*F. prausnitzii*,*Enterobacteriaceae*,*Clostridium*cluster IV,*Bifidobacterium*and*Lactobacillus*group by qPCR. Amplicon size, annealing and fluorescence acquisition temperature are also reported**

**Target microorganism**	**Primer set**	**Sequence (5' to 3')**	**Product size (bp)**	**Annealing temp (°C)**	**Fluorescence acquisition temp (°C)**	**Reference**
*Akkermansia muciniphila*	AM1	CAGCACGTGAAGGTGGGGAC	349	63	88	[31]
	AM2	CCTTGCGGTTGGCTTCAGAT				
*Faecalibacterium prausnitzii*	Fprau223F	GATGGCCTCGCGTCCGATTAG	199	67	85	[32]
	Fprau420R	CCGAAGACCTTCTTCCTCC				
*Enterobacteriaceae*	Eco1457F	CATTGACGTTACCCGCAGAAGAAG	195	63	87	[32]
	Eco1652R	CTCTACGAGACTCAAGCTTGC				
*Clostridium Cl_IV*	S-*-Clos-0561-a-S-17	TTACTGGGTGTAAAGGG	588	60	85	[33]
	S-*-Clept-1129.a-A-17	TAGAGTGCTCTTGCGTA				
*Bifidobacterium*	bif-164	GGGTGGTAATGCCGGATG	523	60	90	[34]
	bif-662	CCACCGTTACACCGGGAA				
*Lactobacillus* group	Lac1	AGCAGTAGGGAATCTTCCA	327	61	85	[35]
	Lac2	ATTYCACCGCTACACATG				

## Results

### Faecal microbiota profile of atopic children and healthy controls

**Figure 1:**
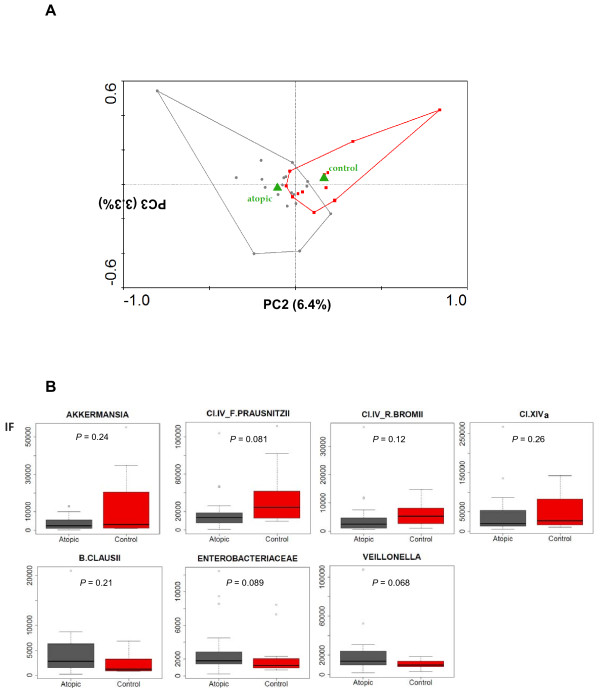
**Analysis of the HTF-Microbi.Array fluorescence signals**. **A:** PCA of the HTF-Microbi.Array fluorescence signals. Atopy or health status were considered as dummy environmental variables (green triangles) and indicated as atopic and control, respectively. Atopic subjects and healthy controls are indicated by gray circles and red squares, respectively. Second and third ordination axes are plotted showing 6.4% and 3.3% of the total variability in the dataset, respectively. **B:** Comparison of the HTF-Microbi.Array probe fluorescence signals between atopics and controls. Only probes showing a different trend between the two groups (*P* < 0.3) are shown.

The faecal microbiota of 19 atopic children and 12 healthy controls living in Italy was characterized by means of the HTF-Microbi.Array platform (Additional files 4 and 5) [24]. Hybridization experiments were performed in two replicates. Pearson’s correlation coefficients ranging from 0.95 and 0.99 were achieved between the two replicates, proving the high reproducibility of the phylogenetic profiles obtained by the HTF-Microbi.Array platform. A PCA of the fluorescence signals from atopics and controls was carried out. The diagnosis of atopy was considered as a dummy environmental variable. As shown in Figure 1A, the principal components PC2 and PC3, which collectively represented only a minor fraction of the total variance (9.7%), resulted in the separation of samples according to the health status. In order to identify the bacterial lineages showing differences in abundance between atopics and controls, probe fluorescence signals obtained from the HTF-Microbi.Array in atopics and controls were compared by box plot analysis (Additional file 6). Probes showing *P* < 0.3 are represented in Figure 1B. Atopic children showed a tendency towards reduction of *A. muciniphilaF. prausnitzii et rel.* and *Ruminococcus bromii et rel.* (*Clostridium* cluster IV), and *Clostridium* cluster XIVa, and were enriched in *EnterobacteriaceaeBacillus clausii* and *Veillonella parvula*.

**Figure 2:**
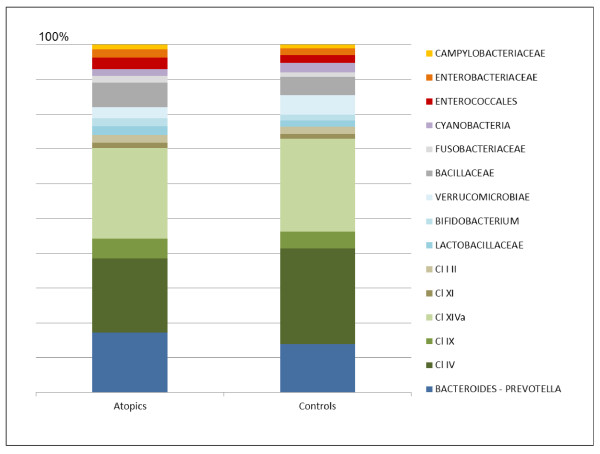
**Relative contribution of the principal intestinal microbial groups in the faecal microbiota of atopics and controls**. For each HTF-Microbi.Array probe, the relative fluorescence contribution was calculated as percentage of the total fluorescence. Sub-probes were excluded. Data represent the mean of the probe relative fluorescence contribution in atopics (n = 19) and controls (n = 12). *P* values derive from a two-sided *t*-test.

On the basis of the HTF-Microbi.Array fluorescence data, the relative contribution of the major phyla in atopics and controls was calculated (Figure 2). At high taxonomic level, atopics and controls showed a comparable overall phylogenetic composition of the faecal microbiota. Indeed, their microbiota resulted largely dominated by *Bacteroidetes* and *Firmicutes,* which together accounted for up to 90% of the faecal microbial community. With a relative abundance ranging from 1 to 5%, *Fusobacteria*, *Actinobacteria* and *Proteobacteria* were sub-dominant components. However, focusing at lower taxonomic level, significant differences in the relative contribution of certain microbial groups were detected. In particular, atopics were characterized by a lower relative contribution of members of the *Clostridium* cluster IV (atopics, 20.9% - controls, 28.7%; *P* = 0.01) and a concomitant relative increase in *Enterobacteriaceae* (atopics, 2.4% - controls, 1.2%; *P* = 0.009) and *Fusobacteria* (atopics, 1.9% - controls, 1.2%; *P* = 0.001).

The abundance of *F. prausnitzii*, *A. muciniphila*, *Enterobacteriaceae, Clostridium* cluster IV, *Bifidobacterium* and *Lactobacillus* group in the faecal microbiota of atopics and controls was investigated by qPCR analysis of the 16 S rRNA gene. As reported in Table 3, respect to healthy controls, atopics were significantly depleted in *F. prausnitzii*, *A. muciniphila* and members of the *Clostridium* cluster *IV*, and tended to be depleted in *Bifidobacterium* and enriched in *Enterobacteriaceae*.

**Table 3:** **qPCR quantification of*F. prausnitzii*,*A. muciniphila*,*Enterobacteriaceae, Clostridium*cluster IV,*Bifidobacterium*and*Lactobacillus*group in the faecal microbiota of atopics and healthy controls**

	**16S rRNA gene copies/μg fecal DNA**		
**Bacterial species/group**	**Atopics**	**Controls**	***P*value**
*Faecalibacterium prausnitzii*	6.17E + 06	2.03E + 07	**0.0014**
*Akkermansia muciniphila*	3.01E + 05	5.03E + 05	**0.0190**
*Enterobacteriaceae*	3.86E + 04	1.19E + 04	0.3500
*Clostridium* cluster IV	4.46E + 06	1.55E + 07	**0.0035**
*Bifidobacterium*	1.08E + 06	1.72E + 06	0.0850
*Lactobacillus* group	3.75E + 02	5.48E + 02	0.6410

For each bacterial species/group, the mean 16S rRNA copy number per μg of faecal DNA is reported.

### Correlation among faecal microbiota, diagnosis of allergy and total IgE

In order to investigate whether the profile of the faecal microbial community of atopics correlated with their allergy profile (Table 1), a PCA of the HTF-Microbi.Array fluorescence signals from atopics was carried out. PCA was performed by considering the types of allergic response as dummy environmental variables. No separation of the atopic children according to the specific diagnosis of rhinitis, asthma, grass pollen sensitization, allergic atopic dermatitis, oral allergy syndrome and cow’s milk allergy was obtained, proving that the atopy-related dysbioses of the faecal microbiota are independent of the specific atopic outcome (data not shown).

**Figure 3:**
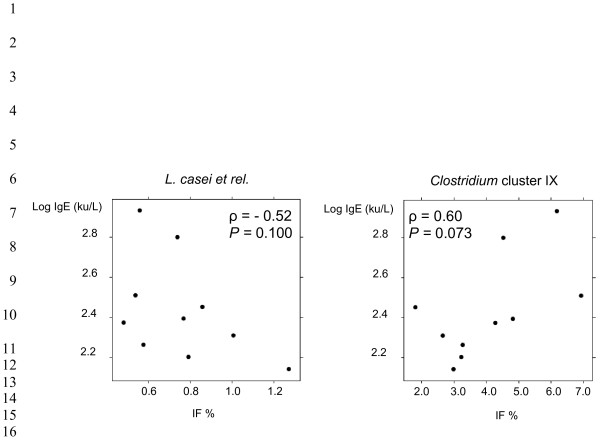
**Spearman rank correlation between total IgE level and the abundance of*L. casei et rel.*and*Clostridium*cluster IX in the stools from a subset of 10 atopic children.**

In a subset of 10 atopy cases with clinical relevance the total serum IgE levels were determined. Total IgE ranged from 138 to 855 ku/L (geometric mean: 326 ku/L), a value above the normal for age [27]. In order to investigate whether in this subset of 10 atopics IgE correlated with the relative abundance of a specific microbial group in the faeces, Spearman rank correlation coefficients between the probe relative fluorescence signals and the IgE levels were calculated. According to our data no significant correlation was determined. However, a tendency towards an inverse correlation with IgE was obtained for *L. casei et rel.* (*ρ* = 0.52; *P* = 0.100), while *Clostridium* cluster IX abundance tended to be positively correlated with total IgE (*ρ* = 0.60; *P* = 0.073) (Figure 3).

## Discussion

In the present paper we combined two culture-independent molecular approaches, HTF-Microbi.Array and qPCR, for a pilot characterization of the atopy-associated dysbiosis of the intestinal microbiota in 19 atopic children living in Italy. At high phylogenetic level both atopics and controls showed a comparable overall microbiota profile where *Firmicutes* and *Bacteroidetes* constituted the two dominant divisions. However, focusing at lower taxonomic level, the intestinal microbiota of atopic children was characterized by a significant depletion in members of the *Clostridium* cluster IV, *F. prausnitzii*, *A. muciniphila* and a corresponding increase of the relative abundance of *Enterobacteriaceae.*

In a case–control DGGE-based study of the faecal microbiota from 20 allergic and 20 non-allergic 5-year-old Estonian children, Stsepetova *et al.*[36] reported a less diverse composition in the faecal microbiota from atopic children but, according to the Authors, no bacterial targets could distinguish infants with or without atopy. However, the DGGE-based approach allowed to consider only the dominant fraction of the intestinal microbiota, remaining blind with respect to the whole phylogenetic complexity of the ecosystem. In an elegant 16 S rDNA pyrosequencing-based dynamic study, Hong *et al.*[37] addressed the differences in the microbiota succession between 3 infants with and 4 without eczema over four time points until 24 months of age. Even if age was shown to be the dominant factor mediating microbiota changes, matched by age eczema infants were characterized by a higher abundance of the enterobacteria *Klebsiella* and *Shigella* as well as *Enterococcus*, while *Bifidobacterium* showed a higher abundance in non-eczema ones. These last data are in general agreement with the intestinal microbiota dysbioses observed in our study.

Although *Bifidobacterium* and *Lactobacillus* have been traditionally indicated as possible protective factors against atopic disease in childhood [16], we did not detect any significant differences in these health-promoting genera between atopics and controls, confirming previous findings reported by Penders *et al.*[3,18]. However, molecular studies at the species level showed a different distribution of the *Bifidobacterium* and *Lactobacillus* species between allergic and non-allergic children [36,38], suggesting a potential species-specific effect of *Bifidobacterium* and *Lactobacillus* in the etiology of atopic disorders.

The atopy-related microbiota dysbioses we depicted in our cohort of 19 children were independent of their peculiar allergic profile. A subset of 10 atopics underwent clinical evaluation of total IgE level and the correlation between IgE and the relative abundance of specific microbial groups in the faeces was explored. Even if no significant correlation was determined, *L. casei et rel.* and *Clostridium* cluster IX tended to be negatively and positively correlated with IgE, respectively. Interestingly, Ogawa *et al.*[39] demonstrated that orally administered *L. casei* was effective in the control of the IgE levels in human allergic reactions and, recently, Schiffer *et al.*[40] reported that *L. casei* could inhibit the effector phase of immune inflammation *in vivo*. Finally, Penders *et al.*[38] showed a decreased risk of atopic dermatitis in children colonized by *L. paracasei*, a member of the *L. casei et rel.* group. Even if these studies may support the tendency towards inverse correlation between *L. casei et rel.* and IgE level we observed in our study, caution must be taken in considering these data since only a low number of children were analyzed.

Characterized by a decrease of the absolute levels of *Clostridium* cluster IV, *F. prausnitzii* and *A. muciniphila*, as well as a corresponding increase in the relative abundance of *Enterobacteriaceae*, the atopy-associated intestinal microbial community we described in this study is depleted in key immunomodulatory members of the human intestinal microbiota and possibly enriched in pro-inflammatory “pathobionts” [41]. By the specific induction of T regs, members of the *Clostridium* cluster IV have been demonstrated to be strategic for maintaining the immune homeostasis [42]. Analogously, providing a vast range of anti-inflammatory effects, *F. prausnitzii* has been considered as a crucial microorganism for gut homeostasis [43]. Finally, *A. muciniphila* is a common member of the human intestinal tract which has been recently associated with a protective/anti-inflammatory role in healthy gut [44]. On the other hand, *Enterobacteriaceae* have been reported to prosper in the context of a host-mediated inflammatory response [45]. Capable to venture more deeply in the mucus layer and establish a close interaction with the epithelial surface, members of *Enterobacteriaceae* concur in the induction of a pro-inflammatory response and further consolidate the host inflammatory status. Thus, similarly to the one characterized in IBD [43,46-48], the atopy-associated microbiota can represent an inflammogenic microbial consortium which can contribute to the severity of the disease [7].

## Conclusion

Atopic children were depleted in specific members of the intestinal microbiota that, capable to orchestrate a broad spectrum of inflammatory and regulatory T cell responses, have been reported as fundamental for the immune homeostasis. The decrease of these key immunomodulatory symbionts in the gastrointestinal tract – as well as the corresponding increase in relative abundance of pro-inflammatory *Enterobacteriaceae* – support the immune deregulation and, in the context of an atopic host, can sustain an inflammatory status throughout the body. Since the atopy-related dysbioses of the intestinal microbiota can contribute to the severity of the disease, atopy treatment may be facilitated by redressing these microbiological unbalances. To this aim, advantages can be taken from the possibility to manipulate the microbiota plasticity with diet or pharmaceutical prebiotics and probiotics. However, the phylogenetic resolution of the data reported in our study needs to be implemented by deep 16 S rDNA sequencing. Moreover, metatranscriptomic studies can be carried out. Linking the phylogenetic structure of the intestinal microbiota with its specific functional activities, the metatranscriptomic characterization of the intestinal microbiota in atopic children could reveal the possible pathogenic mechanisms behind the atopy-related microbiota dysbioses.

## Abbreviations

A: Asthma; AD: Allergic Atopic Dermatitis; CMA: Cow’s Milk Allergy; GPS: Grass Pollen Sensitization; HTF-Microbi.Array: High Taxonomic Fingerprint-Microbi.Array; IBD: Inflammatory Bowel Diseases; IF: Fluorescence Intensities; KOALA: Is (in Dutch) an acronym for Child Parent and health, Lifestyle and Genetic constitution; LDR-UA: Ligase Detection Reaction-Universal Array; OAS: Oral Allergy Syndrome; PCA: Principal Component Analysis; PC1 2 and 3: Principal Components 1, 2 and 3; RC: Rhinitis.

## Authors’ contributions

MC conceived and designed the experiments, analyzed the data and wrote the first draft of the paper. SR and ST performed faecal microbial DNA extraction, 16 S rDNA amplification and purification, qPCR bacterial quantifications and PCA analysis. MS, CC, GDB performed all the HTF-Microbi.Array hybridization experiments and data analysis. RM, GR and AP enrolled subjects and performed skin prick test and IgE determination. PB conceived and designed the experiments. All authors read and approved the final manuscript.

## Supplementary Material

Additional file 1: Phylogenetically related groups target of the HTF-Microbi.Array. Click here for file

Additional file 2: Probe specificity tests for *Akkermansia muciniphila*. Data refer to independent duplicates obtained using 50 fmol of purified 16 S rRNA PCR product. X axis shows the ZipCode for each probe pair; in both figures, “1B” represents the ZipCode associated to *A. muciniphila*. Y axis shows the average fluorescence intensities (IF) for each probe pair. Fluorescence between the two replicates was not normalized. Blue stars over the fluorescence bars indicate the probes that gave a positive response with *P* <0.01. Red dots indicate that one or two replicates out of four for each ZipCode were excluded because of having an IF 2.5-fold above or below the average of the spots. Click here for file

Additional file 3: HTF-Microbi.Array probe list. Sequences (5’ - > 3’) for both discriminating (DS) and common probe (CP) are reported, as well as major thermodynamic parameters [melting temperature (Tm), length (bp), number of degenerated bases (Deg)]. Click here for file

Additional file 4: HTF-Microbi.Array raw fluorescence data obtained from the analysis of faecal stools from 19 atopic children (A) and 12 healthy controls (C). Click here for file

Additional file 5: Layout of the HTF-Microbi.Array and complete ZipCode sequences. Click here for file

Additional file 6: Box plots of the HTF-Microbi.Array fluorescence signals from atopics and controls. *P* values corresponding to the difference in fluorescence response between the two groups are indicated for each probe. Click here for file
