# Supplementary figures and images for: Unbalance of intestinal microbiota in atopic children
Source: BMC Microbiol. 2012 Jun 6;12:95. doi: 10.1186/1471-2180-12-95 (PMC3404014; doi:10.1186/1471-2180-12-95)

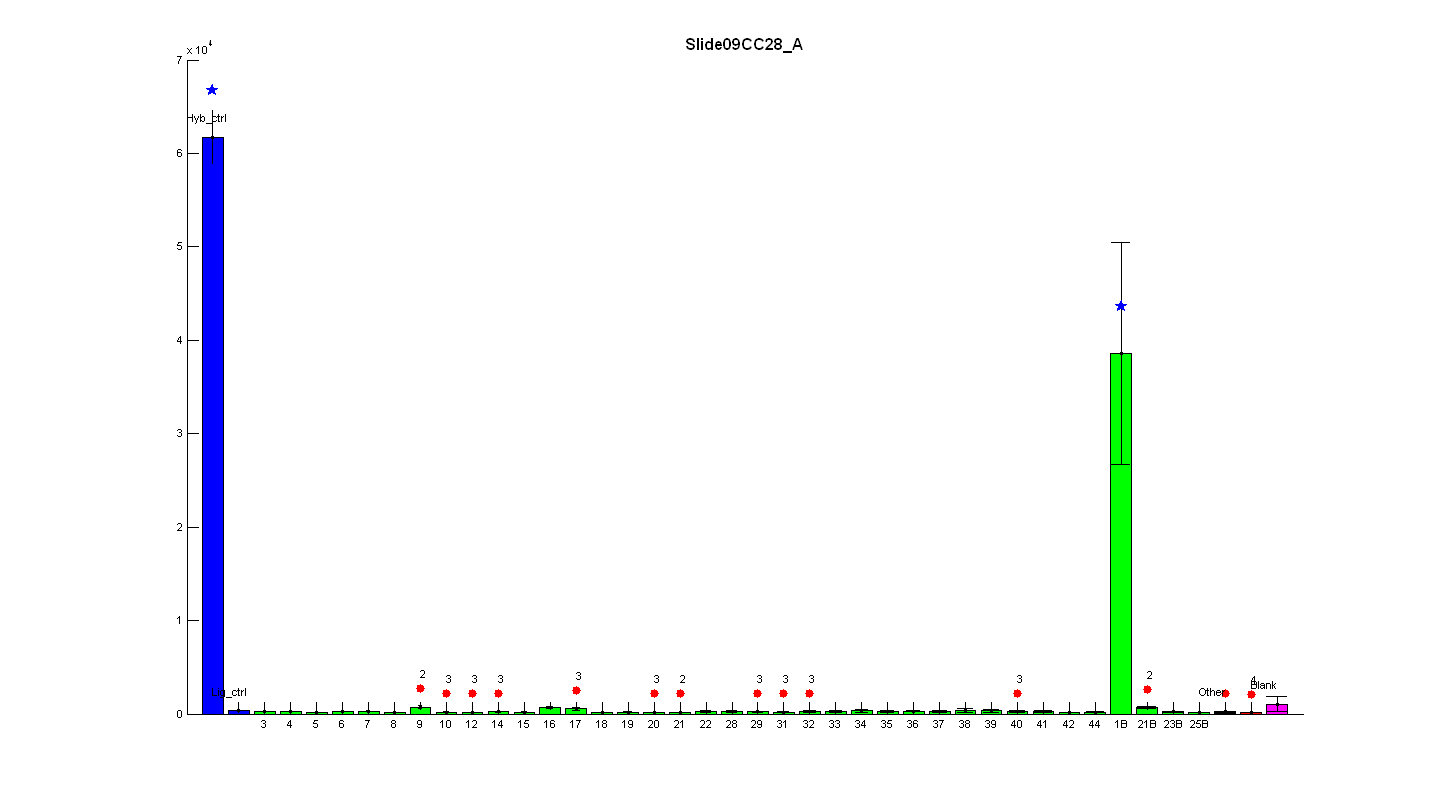

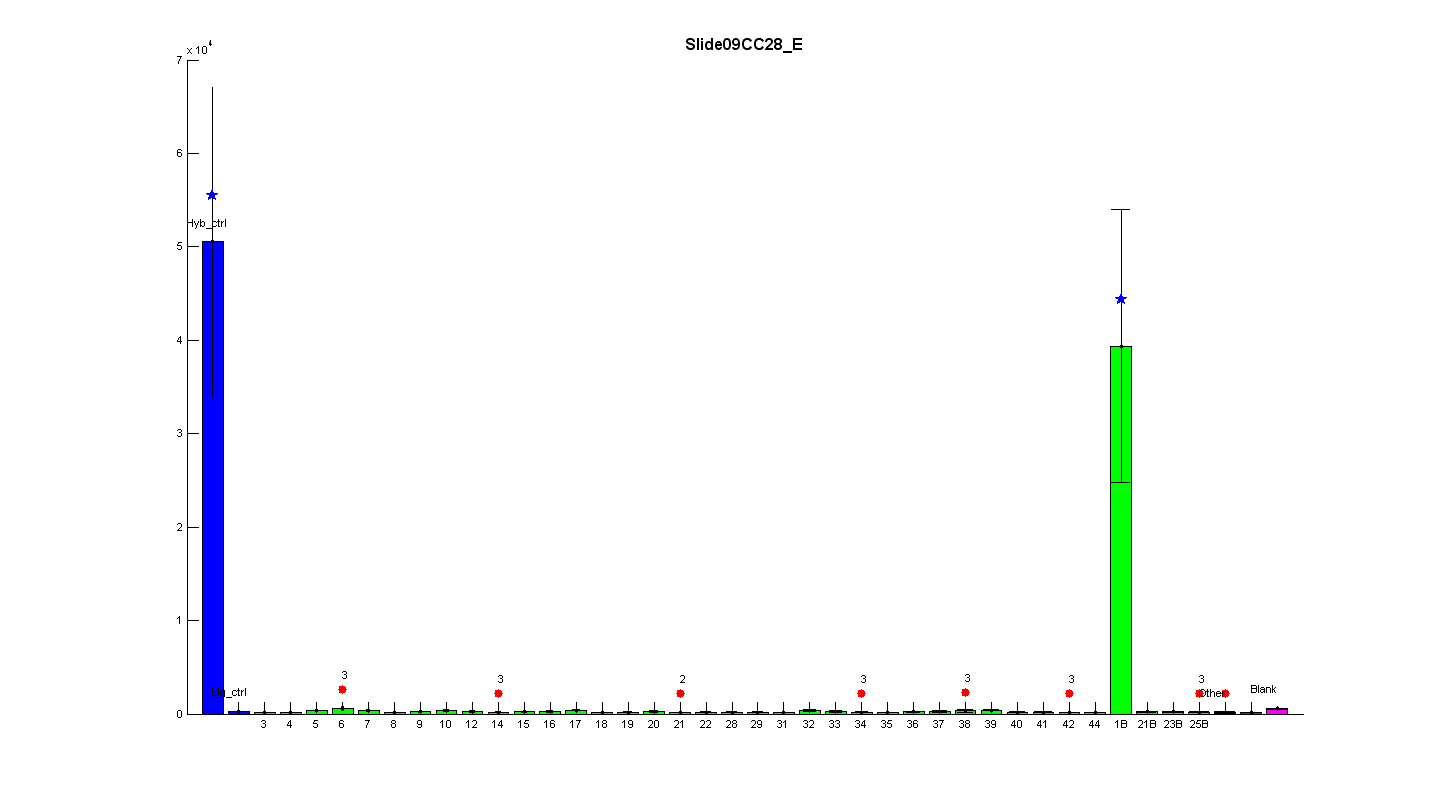


**(B)**

**(A)**

Supplement: Additional file 2: — Probe specificity tests for Akkermansia muciniphila. Data refer to independent duplicates obtained using 50 fmol of purified 16 S rRNA PCR product. X axis shows the ZipCode for each probe pair; in both figures, “1B” represents the ZipCode associated to A. muciniphila. Y axis shows the average fluorescence intensities (IF) for each probe pair. Fluorescence between the two replicates was not normalized. Blue stars over the fluorescence bars indicate the probes that gave a positive response with P <0.01. Red dots indicate that one or two replicates out of four for each ZipCode were excluded because of having an IF 2.5-fold above or below the average of the spots. [file 1471-2180-12-95-S2.doc]
