# Supplementary material for: Unbalance of intestinal microbiota in atopic children
Source: BMC Microbiol. 2012 Jun 6;12:95. doi: 10.1186/1471-2180-12-95 (PMC3404014; doi:10.1186/1471-2180-12-95)
Supplement: Additional file 3: — HTF-Microbi.Array probe list. Sequences (5’ - > 3’) for both discriminating (DS) and common probe (CP) are reported, as well as major thermodynamic parameters [melting temperature (Tm), length (bp), number of degenerated bases (Deg)]. [file 1471-2180-12-95-S3.doc]

| **Seq Name** | **Zip code** | **Discriminating oligo (DS)** | **Common probe (CP)** | **DS**  **bp** | **CP**  **bp** | **Tm DS** | **Tm CP** | **Deg DS** | **Deg CP** |
| --- | --- | --- | --- | --- | --- | --- | --- | --- | --- |
| B. longum | 3 | GTATGGGATGGGGTCGCGTCCTATCAGCTTGA**C** | GGCGGGGTAACGGCCNACCGTGGCT | 33 | 25 | 68.1 | 68.3 | 0 | 1 |
| Yersinia | 4 | GAGTAGGGCTACACACGTGCTACAATGGCAGATACAAAG**T** | GAAGCGAACTCGCGAGAGCAAGCGGACC | 40 | 28 | 67.6 | 67.2 | 0 | 0 |
| Proteus | 5 | GGTCTTGAACCGTGGCTTCTGGAGCTAACGCGTTAA**A** | TCGACCGCCTGGGGAGTACGGCCGC | 37 | 25 | 67.8 | 69.2 | 0 | 0 |
| Campylobacter | 6 | GCTAGTTGGTRAGGTAATGGCTTACCAAGGCTATGACGCWTA**A** | CTGGTCTGAGAGGATGATCAGTCACACTGGAACTGAG | 43 | 37 | 67.9 | 67.8 | 2 | 0 |
| B. cereus | 7 | ACTCTGTTGTTAGGGAAGAACAAGTGCTAGTTGAATAAG**C** | TGGCACCTTGACGGTACCTAACCAGAAAGCCACGG | 40 | 35 | 64.5 | 69.1 | 0 | 0 |
| B. subtilis | 8 | GCAGCGAAACCGCGAGGTTAAGCCAATCCCA**C** | AAATCTGTTCTCAGTTCGGATCGCAGTCTGCAACTCGA | 32 | 38 | 68.2 | 66.6 | 0 | 0 |
| E. faecalis | 9 | GGAAGTACAACGAGTCGCTAGACCGCGAGGTCA**T** | GCAAATCTCTTAAAGCTTCTCTCAGTTCGGATTGCAGGCTG | 34 | 41 | 68.0 | 67.5 | 0 | 0 |
| E. faecium | 10 | CGCTTCTTTTTCCACCGGAGCTTGCTCCACC**G** | GAAAAAGARGAGTGGCGAACGGGTGAGTAACACGTGG | 32 | 37 | 68.2 | 68.3 | 0 | 1 |
| L. casei | 12 | GGGTCGTAAAACTCTGTTGTTGGAGAAGAATGGTCGG**C** | AGAGTAACTGTTGTCGGCGTGACGGTATCCAACCAG | 38 | 36 | 67.7 | 67.9 | 0 | 0 |
| L. salivarius | 14 | GTGAAATGCGTAGATATATGGAAGAACACCAGTGGCGAA**A** | GCGGCTCTCTGGTCTGTAACTGACGCTGAGG | 40 | 31 | 65.5 | 68.3 | 0 | 0 |
| Fusobacterium | 15 | GGGGAAGCCAGCYTACTGGACAGATACTGACGCTRA**A** | GCGCGAAAGCGTGGGTAGCAAACAGGATTAGATACC | 37 | 36 | 68.9 | 67.9 | 2 | 0 |
| Bateroides /  Prevotella | 16 | CATTAAGYATYCCACCTGGGGAGTACGCCGGCAA**C** | GGTGAAACTCAAAGGAATTGACGGGGGCCCGC | 35 | 32 | 69.1 | 68.2 | 2 | 0 |
| C. perfringens | 17 | CTACACTTGACATCCCTTGCATTACTCTTAATCGAGGAA**A** | TCCCTTCGGGGACAAGGTGACAGGTGGTGCAT | 40 | 32 | 64.5 | 68.2 | 0 | 0 |
| C. difficile | 18 | GAACGCTGGCGGCGTGCCTAACACATGCAAGT**T** | GAGCGATTTACTTCGGTAAAGAGCGGCGGACGG | 33 | 33 | 68.1 | 68.1 | 0 | 0 |
| E. rectale | 19 | CATTGCTTCTCGGTGCCGTCGCAAACGCA**G** | TAAGTATTCCACCTGGGGAGTACGTTCGCAAGAATGAAACTC | 30 | 42 | 67.1 | 67.4 | 0 | 0 |
| Veillonella | 20 | GGTGGGAACTCATGAGAGACTGCCGCAGACAA**T** | GCGGAGGAAGGCGGGGATGACGTCAAATC | 34 | 29 | 66.9 | 67.2 | 0 | 0 |
| Bifidobacteriaceae | 25B | TAGGGGAGACTGGAATTCCCGGTGTAACGGTGGAATG**T** | GTAGATATCGGGAAGAACACCAATGGCGAAGGCAGGTCT | 38 | 39 | 68.8 | 68.7 | 0 | 0 |
| Enterobacteriaceae | 23B | GGGACCTTCGGGCCTCTTGCCATCGGATG**T** | GCCCAGATGGGATTAGCTWGTWGGTGGGGTAACG | 30 | 34 | 68.5 | 68.0 | 0 | 2 |
| Lactobacillaceae | 21B | AAGAACACCAGTGGCGAAGGCGGCTSTCTGG**T** | CTGTAACTGACGCTGAGGCTCGAAAGCATGGGTAGC | 32 | 36 | 68.2 | 69.0 | 1 | 0 |
| Clostridium XIVa | 22 | CCGCGTGAGYGAAGAAGTATTTCGGTATGTAAAGCTCT**A** | TCAGCAGGGAAGAWAATGACGGTACCTGACTAAGAAGCNC | 39 | 40 | 66.1 | 68.1 | 1 | 2 |
| B. clausii | 32 | CCTAGAGATAGGGCTTTCCCCTTCGGGGGACA**A** | AGTGACAGGTGGTGCATGGTTGTCGTCAGCTCG | 33 | 33 | 68.1 | 68.1 | 0 | 0 |
| L. plantarum | 33 | CTACAATGGATGGTACAACGAGTTGCGAACTCGCGAG**A** | GTAAGCTAATCTCTTAAAGCCATTCTCAGTTCGGATTGTAGGCTG | 38 | 45 | 67.7 | 67.3 | 0 | 0 |
| Clostridium I / II | 35 | GCGTAAAGGGWGCGTAGGYGGATNTTTAAGTGRGATGTGAAAT**A** | CCCGGGCTYAACYTGGGTGCTGCATTYCAAAC | 44 | 32 | 67.8 | 67.6 | 4 | 3 |
| Clostridium XI | 36 | CGATGAAGGCCTTCGGGTCGTAAAGCTCTGTC**C** | TAARGGAAGAWAATGACGGTACYTTAGGAGGAAGCCCCG | 33 | 39 | 68.1 | 67.6 | 0 | 3 |
| Clostridium IX | 37 | GAGCGAACGGGATTAGATACCCCGGTAGTCCT**G** | GCCGTAAACGATGGRTACTAGGTGTAGGAGGTATCG | 33 | 36 | 68.1 | 67.3 | 0 | 1 |
| Ruminococcus bromii  (Clostridium IV ) | 38 | GAACCTTACCAGGTCTTGACATCCAACTAACGAAGTAGAGATRC**A** | TTAGGTGCCCTTCGGGGAAAGKTGAGACAGGTG | 45 | 33 | 67.7 | 67.5 | 1 | 1 |
| Ruminococcus albus  (Clostridium IV ) | 39 | GAATTCCTAGTGTAGCGGTGAAATGCGTAGATATTAGGAGGAACA**T** | CAGTGGCGAAGGCGGCTTACTGGGCTTTAACTG | 46 | 33 | 67.2 | 68.1 | 0 | 0 |
| Faecalibacterium prausnitzii  (Clostridium IV ) | 40 | GTAAAGGGAGCGCAGGCGGGANGGCAAGT**T** | GGAAGTGAAATCTATGGGCTCAACCCATGAACTGCTTTCAAAAC | 30 | 44 | 67.8 | 67.3 | 1 | 0 |
| Oscillospira guillermondii  (Clostridium IV ) | 41 | GGCYTTCGGGTTGTAAACTTCTTTTAAGGGGGAAGARCAGA**A** | GACGGTACCCCTTGAATAAGCCACGGCTAACTACG | 42 | 35 | 67.4 | 67.9 | 2 | 0 |
| Cyanobacteria | 42 | CCCAGACTCCTACGGGAGGCAGCAGT**G** | GGGAATTTTCCGCAATGGGCGAAAGCCTGACGG | 27 | 33 | 67.3 | 68.1 | 0 | 0 |
| Akkermansia | 1B | GTGCCAGCAGCCGCGGTAATACAGAGGTC**T** | CAAGCGTTGTTCGGAATCACTGGGCGTAAAGCGTG | 30 | 35 | 67.1 | 67.9 | 0 | 0 |
