# Supplementary material for: Unbalance of intestinal microbiota in atopic children
Source: BMC Microbiol. 2012 Jun 6;12:95. doi: 10.1186/1471-2180-12-95 (PMC3404014; doi:10.1186/1471-2180-12-95)
Supplement: Additional file 6: — Box plots of the HTF-Microbi.Array fluorescence signals from atopics and controls. P values corresponding to the difference in fluorescence response between the two groups are indicated for each probe. [file 1471-2180-12-95-S6.pdf]

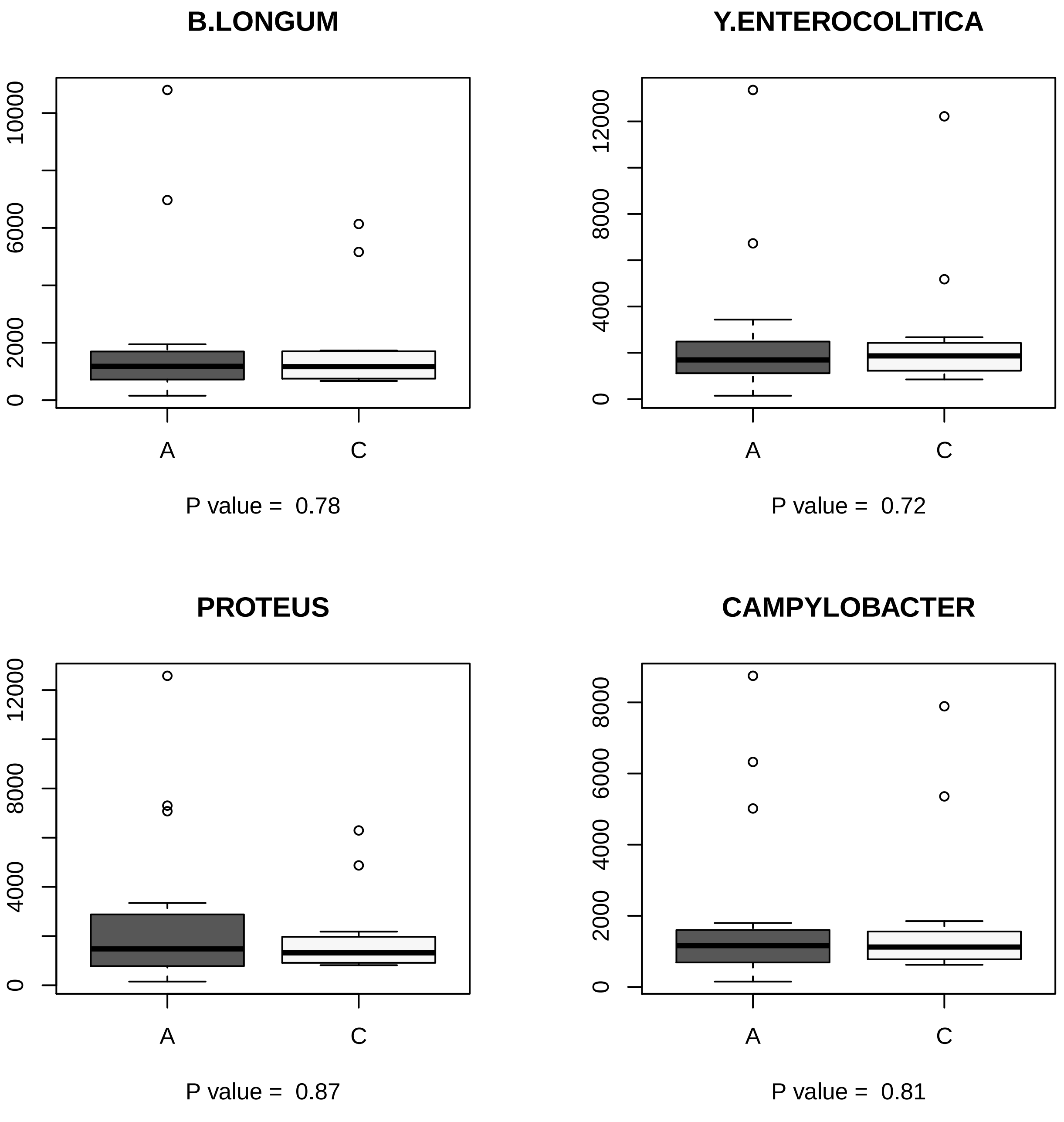
B.LONGUM
0
2000
6000
10000
A
C
P value = 0.78
Y.ENTEROCOLITICA
0
4000
8000
12000
A
C
P value = 0.72
PROTEUS
0
4000
8000
12000
A
C
P value = 0.87
CAMPYLOBACTER
0
2000
4000
6000
8000
A
C
P value = 0.81

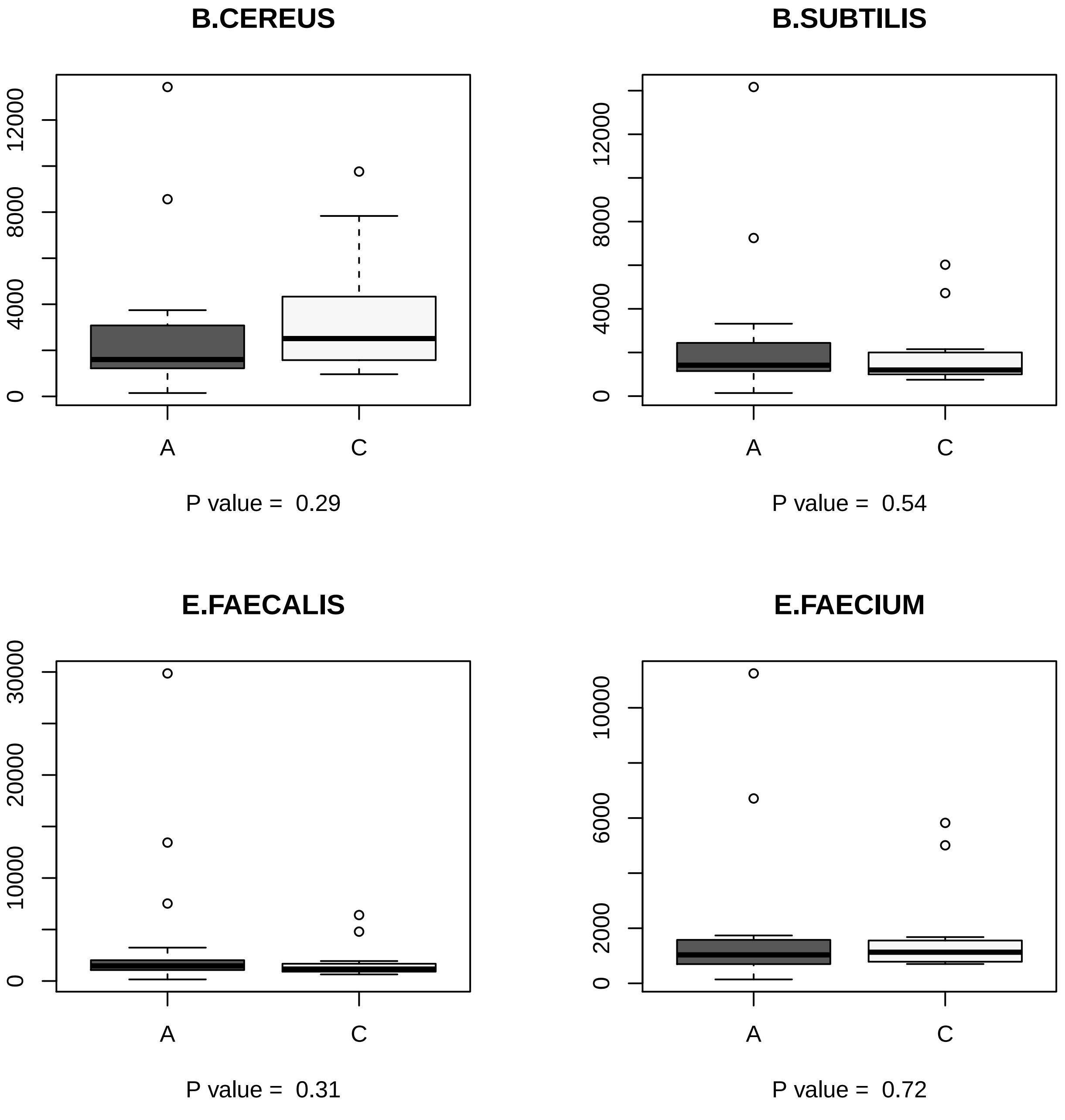
B.CEREUS
0
4000
8000
12000
A
C
P value = 0.29
B.SUBTILIS
0
4000
8000
12000
A
C
P value = 0.54
E.FAECALIS
0
10000
20000
30000
A
C
P value = 0.31
E.FAECIUM
0
2000
6000
10000
A
C
P value = 0.72

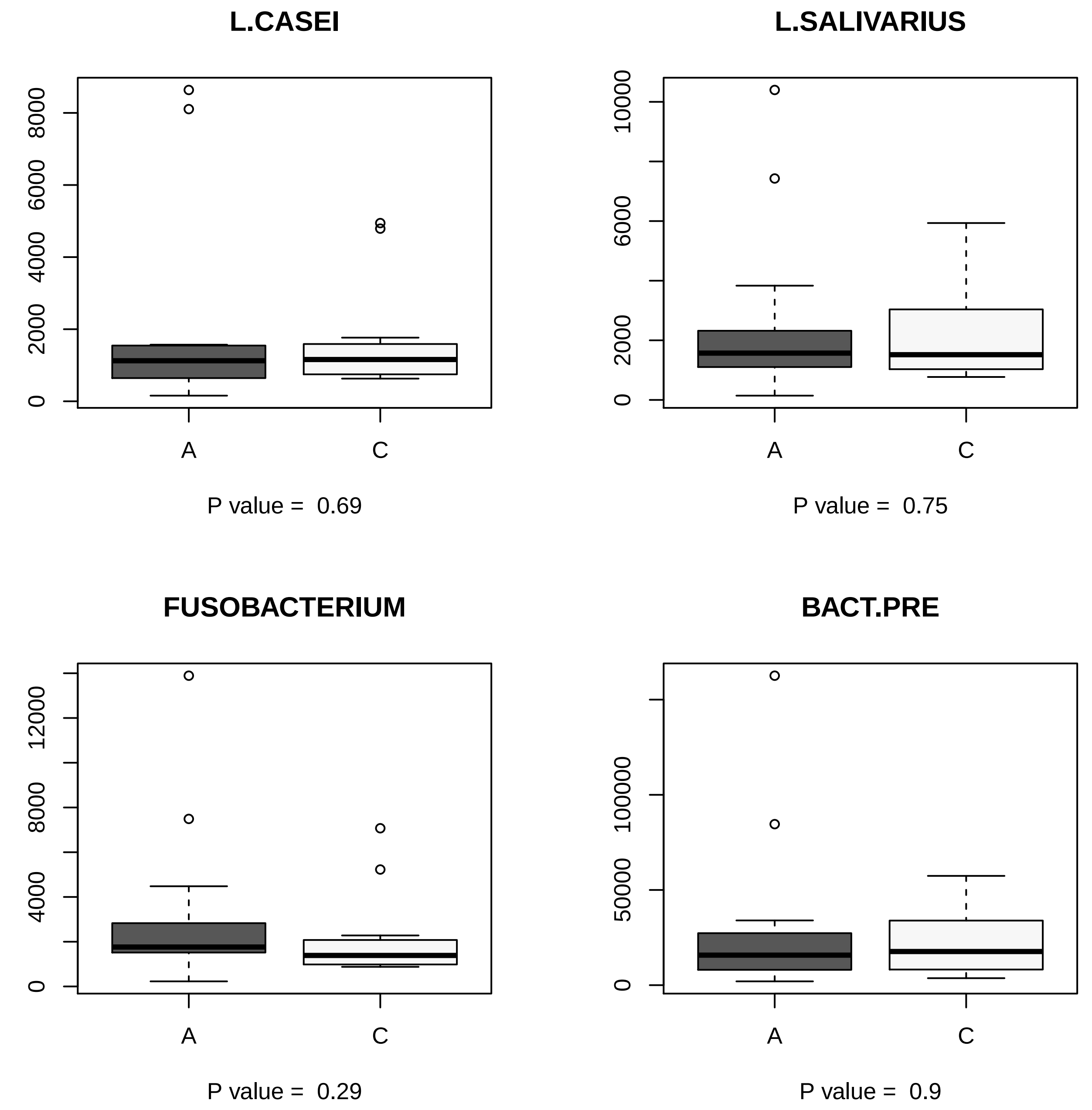
L.CASEI
0
2000
4000
6000
8000
A
C
P value = 0.69
L.SALIVARIUS
0
2000
6000
10000
A
C
P value = 0.75
FUSOBACTERIUM
0
4000
8000
12000
A
C
P value = 0.29
BACT.PRE
0
50000
100000
A
C
P value = 0.9

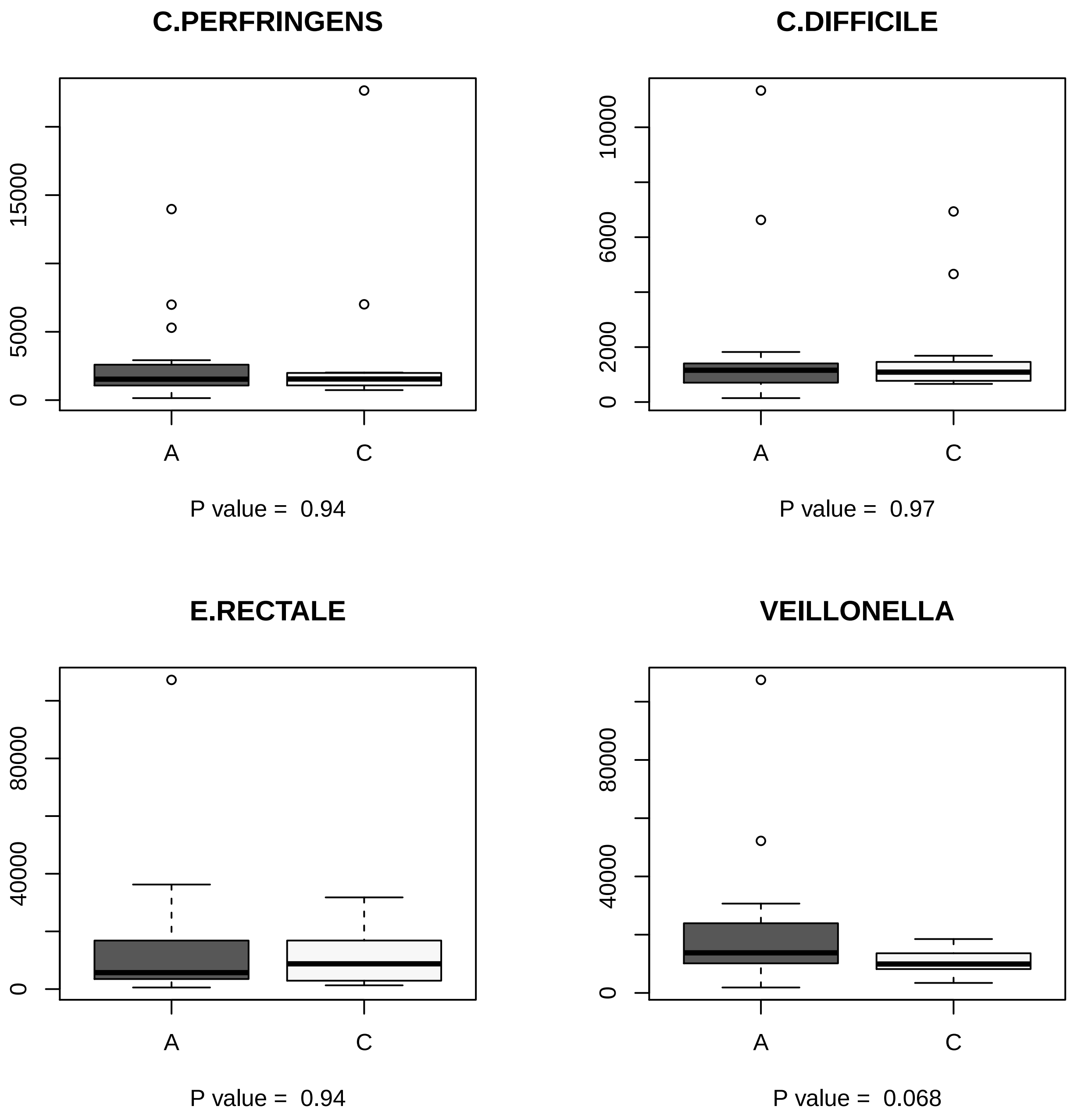
C.PERFRINGENS
0
5000
15000
A
C
P value = 0.94
C.DIFFICILE
0
2000
6000
10000
A
C
P value = 0.97
E.RECTALE
0
40000
80000
A
C
P value = 0.94
VEILLONELLA
0
40000
80000
A
C
P value = 0.068

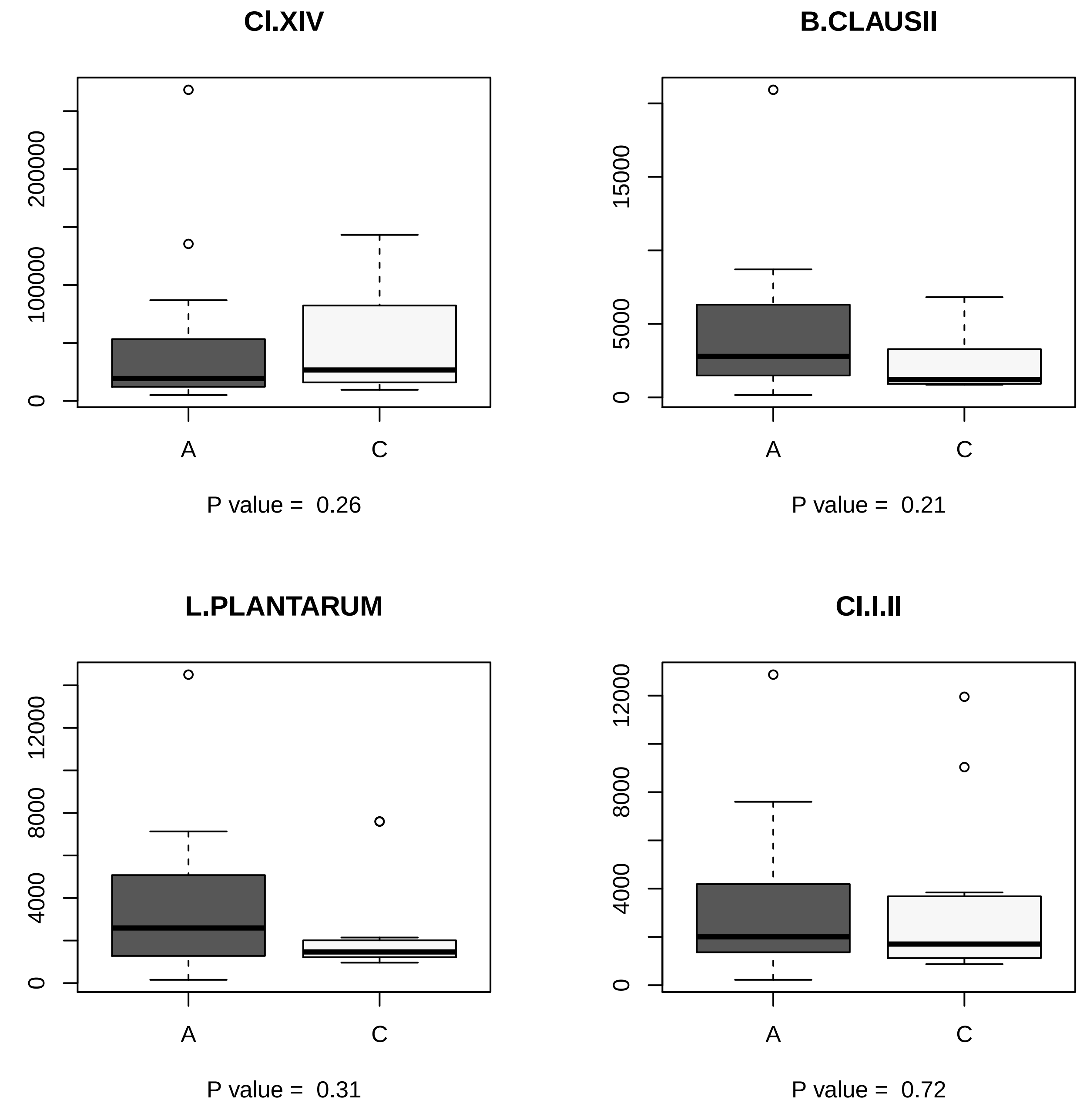
Cl.XIV
0
100000
200000
A
C
P value = 0.26
B.CLAUSII
0
5000
15000
A
C
P value = 0.21
L.PLANTARUM
0
4000
8000
12000
A
C
P value = 0.31
Cl.I.II
0
4000
8000
12000
A
C
P value = 0.72

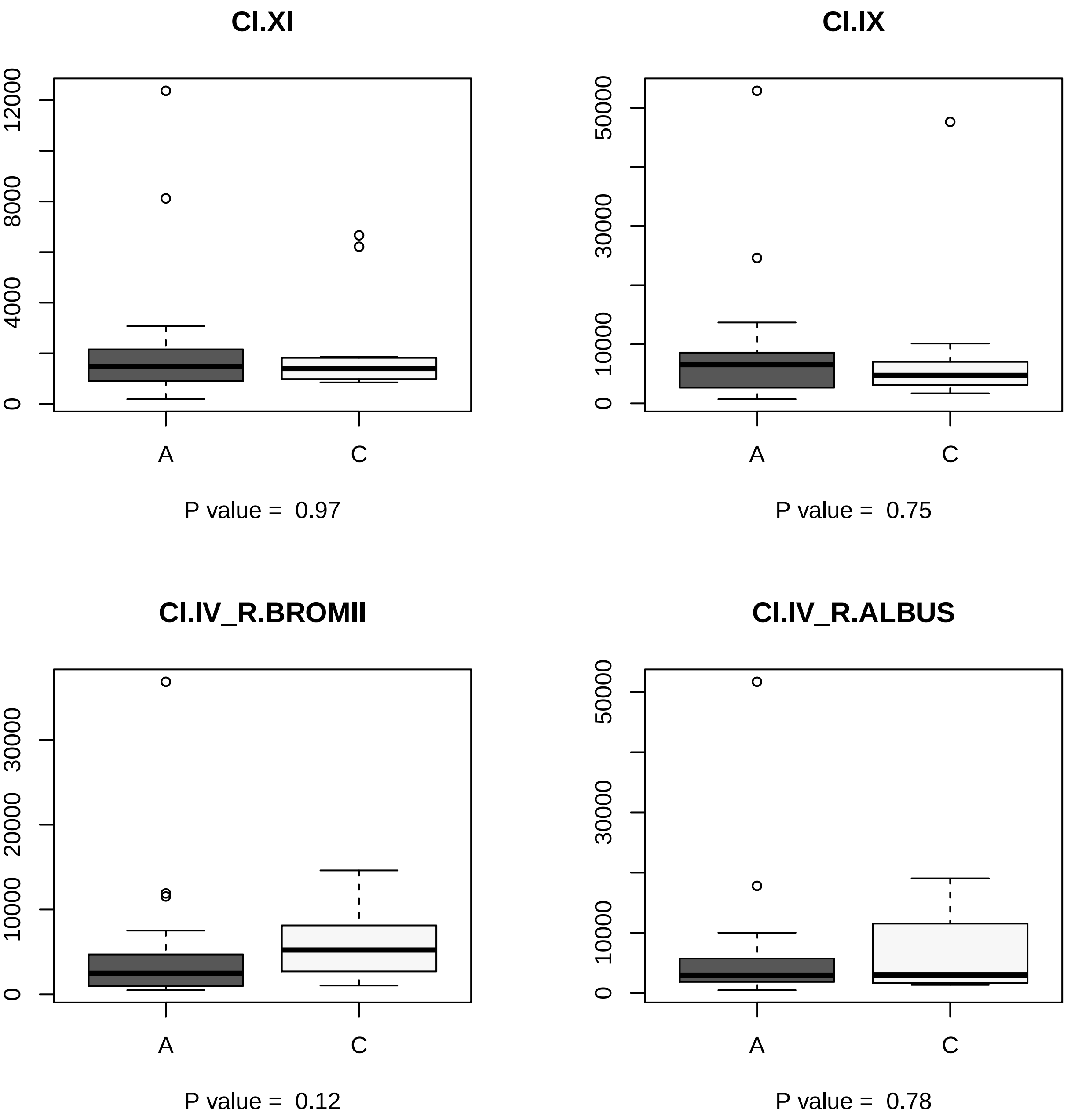
Cl.XI
0
4000
8000
12000
A
C
P value = 0.97
Cl.IX
0
10000
30000
50000
A
C
P value = 0.75
Cl.IV_R.BROMII
0
10000
20000
30000
A
C
P value = 0.12
Cl.IV_R.ALBUS
0
10000
30000
50000
A
C
P value = 0.78

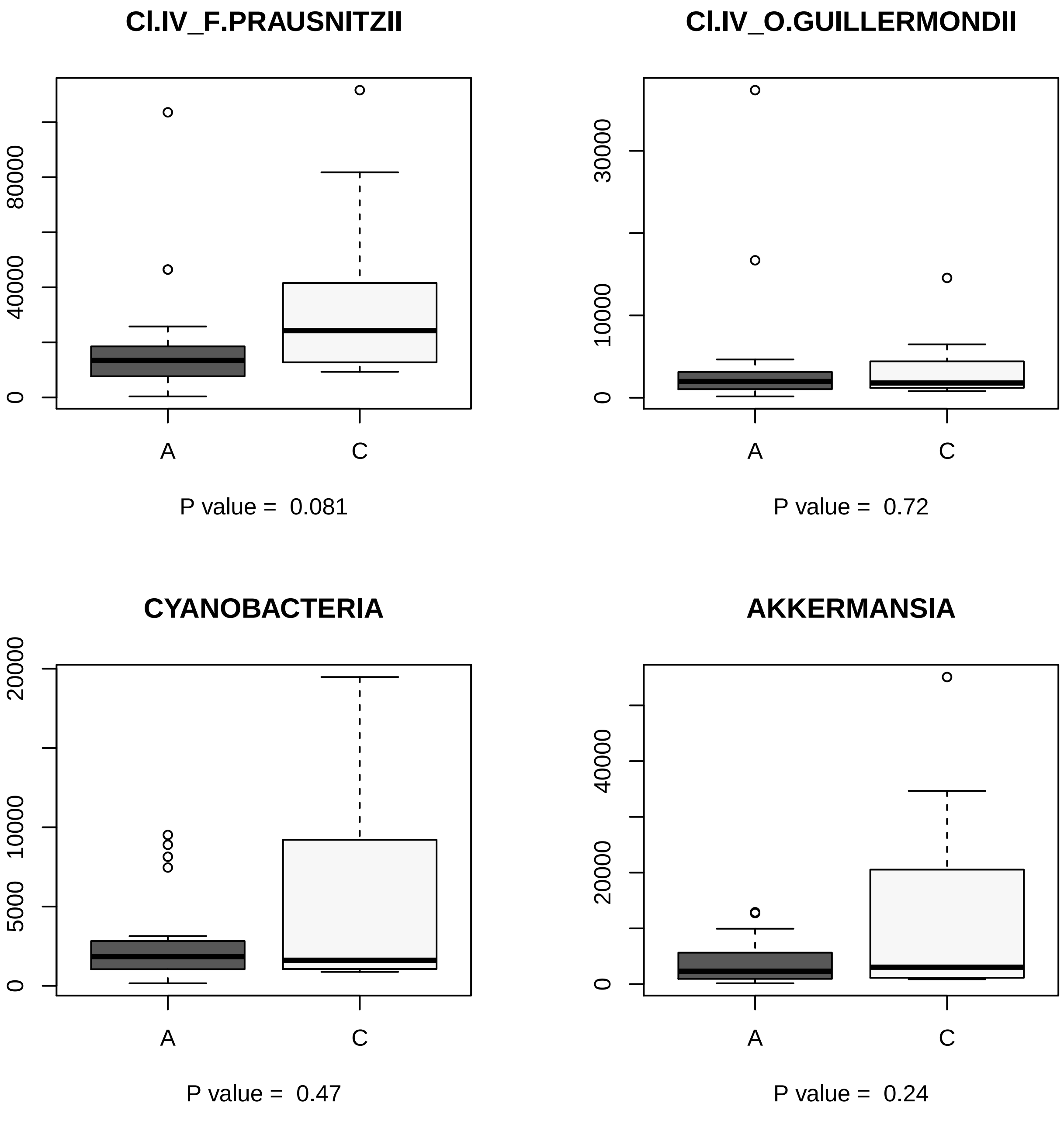
Cl.IV_F.PRAUSNITZII
0
40000
80000
A
C
P value = 0.081
Cl.IV_O.GUILLERMONDII
0
10000
30000
A
C
P value = 0.72
CYANOBACTERIA
0
5000
10000
20000
A
C
P value = 0.47
AKKERMANSIA
0
20000
40000
A
C
P value = 0.24

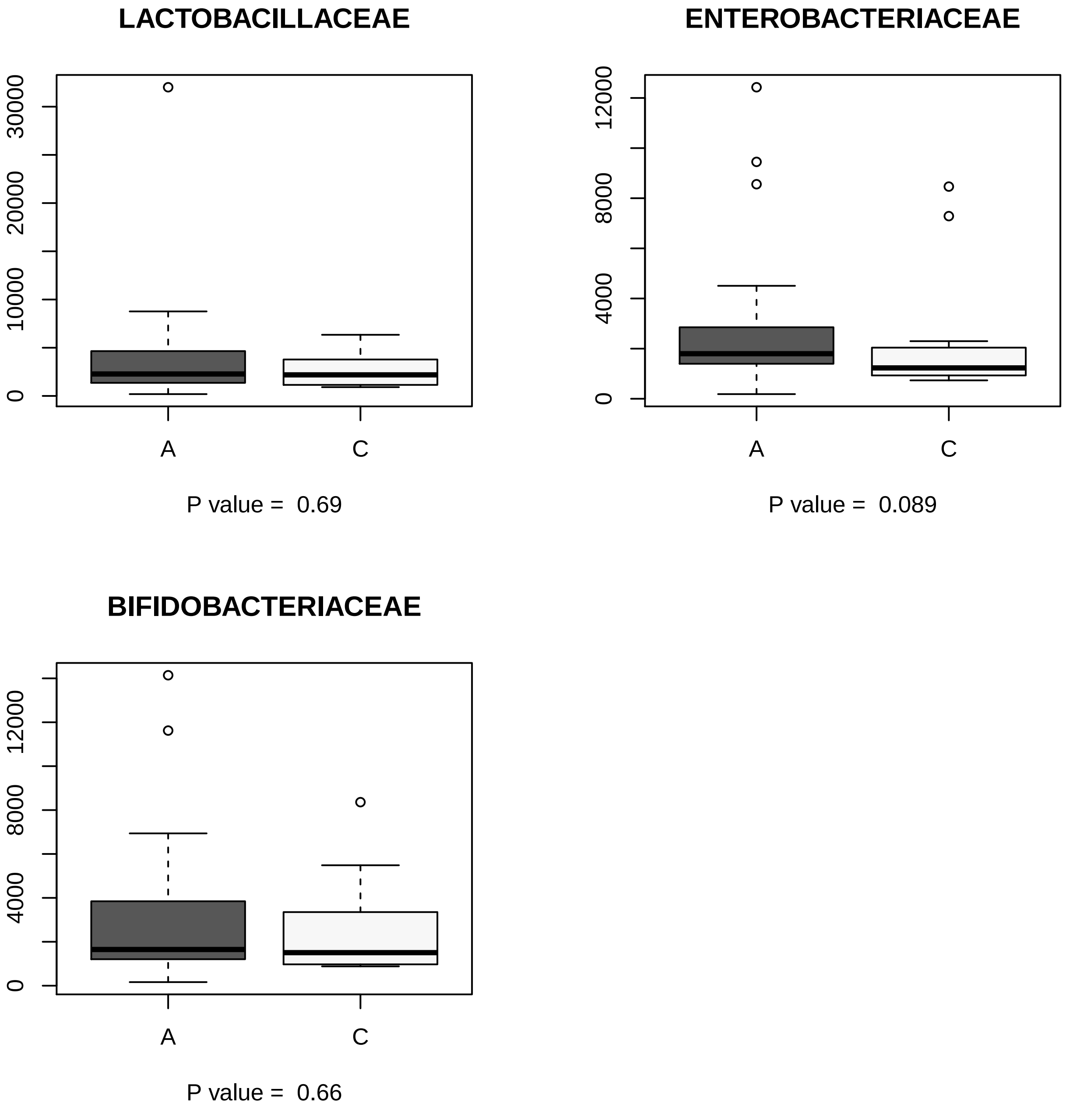
LACTOBACILLACEAE
30000
20000
10000
0
A
C
P value = 0.69
ENTEROBACTERIACEAE
12000
8000
4000
0
A
C
P value = 0.089
BIFIDOBACTERIACEAE
12000
8000
4000
0
A
C
P value = 0.66
